# Redes vivas en salud en la pandemia de covid-19: evaluación del curso de formación multiprofesional “Nós da Linha de Frente” en Manaus, Amazonas, Brasil

**DOI:** 10.18294/sc.2024.4650

**Published:** 2024-02-06

**Authors:** Munique Therense, André Luiz Machado das Neves

**Affiliations:** 1 Doctora en Saúde Coletiva. Profesora Adjunta, Universidade do Estado do Amazonas, Manaus, Brasil. mtpontes@uea.edu.br Universidade do Estado do Amazonas Universidade do Estado do Amazonas Manaus Brazil mtpontes@uea.edu.br; 2 Doctor en Saúde Coletiva; Profesor Adjunto, Universidade do Estado do Amazonas, Manaus, Brasil. almachado@uea.edu.br Universidade do Estado do Amazonas Universidade do Estado do Amazonas Manaus Brazil almachado@uea.edu.br

**Keywords:** Muerte, Duelo, Evaluación Educativa, Actitud Frente a la Muerte, Brasil, Death, Grief, Educational Measurement, Attitude to Death, Brazil

## Abstract

Este artículo presenta la evaluación del curso de intervención terapéutica en situaciones de pérdida traumática y duelo, ofrecido en línea por la Universidade do Estado do Amazonas, entre abril y agosto de 2021, para profesionales voluntarios. Se aplicó un cuestionario semiestructurado con preguntas abiertas y cerradas. El análisis de las 55 respuestas de profesionales voluntarios y 14 respuestas de personas atendidas se basó en el modelo de evaluación Kirkpatrick. Los resultados muestran el acierto teórico y práctico del curso, con una evaluación positiva en los niveles de reacción, aprendizaje, comportamiento y resultados, en la que se destacó el trabajo colectivo y la motivación de los disertantes del curso. Los principales contenidos aprehendidos fueron temas relacionados con el cuidado de la muerte y el duelo. Las conductas derivadas del curso se encuadran en los campos afectivo-social y técnico-profesional. En cuanto al impacto, hubo un predominio de la satisfacción de las personas atendidas, así como identificación de cambios provocados por la formación.

## Abstract (en)

This article presents the evaluation of the therapeutic intervention course in situations of traumatic loss and grief, offered online by the Universidade do Estado do Amazonas, between April and August 2021, for volunteer professionals. A semi-structured questionnaire with open and closed questions was administered. The analysis of the 55 responses from volunteer professionals and 14 responses from individuals who received the intervention was based on the Kirkpatrick evaluation model. The results demonstrate the theoretical and practical success of the course, with a positive assessment at the reaction, learning, behavior, and results levels, emphasizing collective work and the motivation of the course speakers. The main acquired knowledge pertained to topics related to death and grief care. The behaviors resulting from the course fall within the affective-social and technical-professional domains. Regarding impact, there was a predominance of satisfaction among the individuals who received the intervention, as well as identification of changes brought about by the training.

## INTRODUCCIÓN

El inicio de la segunda ola de la crisis sanitaria de la pandemia de covid-19 en el estado de Amazonas, en enero de 2021, fue uno de los capítulos más emblemáticos del contexto pandémico nacional: la crisis de escasez de oxígeno en el norte de Brasil. Las imágenes de familias haciendo fila para obtener cilindros de gas llenos de oxígeno llenaron los titulares, mostrando el colapso de los sistemas de salud tanto públicos como privados en la región.

Este escenario impulsó iniciativas de artistas, profesionales de la salud y una serie de acciones individuales, asociativas, de movimientos e institucionales para ofrecer servicios voluntarios. Se vieron, por ejemplo, campañas de donación de cilindros de oxígeno, recaudación de recursos financieros y suministros para hospitales, además de intervenciones y formación en salud.

Por definición, el voluntariado se refiere a la prestación de servicios dirigidos a la sociedad, derivados de la motivación, el estímulo o el deseo de ayudar al prójimo, sin fines de lucro, con la satisfacción personal y la remuneración abstracta como puntos de partida^1^.

Entre las iniciativas observadas durante la pandemia de covid-19, el presente estudio toma como objeto de análisis la formación del Curso Básico de Intervención Terapéutica en Situaciones de Pérdida Traumática y Duelo, desarrollado por el Proyecto “Nós da Linha de Frente II”. Esta segunda edición del proyecto de extensión “Nós da Linha de Frente” fue un esfuerzo de profesores de la Universidade do Estado do Amazonas (UEA), destinado a dirigir el voluntariado disperso hacia las personas que necesitaban cuidados en medio del escenario pandémico.

Originalmente diseñado en 2020, en un formato dirigido exclusivamente a los profesionales que trabajaban en la primera línea de combate contra la covid-19^2^, la reedición en 2021 tuvo como objetivo reunir los servicios voluntarios ofrecidos por profesionales de la salud para articularlos y conectarlos con la población amazonense.

Su estructura se basó en la organización de alianzas con instituciones públicas y privadas de Brasil, así como con profesionales de proyección nacional en el ámbito académico, que aceptaron formar parte de una red de solidaridad técnicamente competente para la promoción del cuidado y apoyo a la población de Amazonas y otros estados brasileños.

Esta red de cuidado se consolidó como una red viva en salud, es decir, brindó atención más allá de lo establecido formalmente, siendo un movimiento dinámico y constantemente abierto a diversas modalidades de servicios, reconfigurándose y encontrando nuevos flujos que permitieron su crecimiento a partir de las conexiones que se produjeran durante el proceso^3^.

Pensar en estas redes vivas de salud dentro del territorio amazónico asumió una importancia aún mayor en la pandemia, ya que con relación al acceso de las poblaciones amazónicas se han importado modelos estructurales de salud que, en su mayoría, no dialogan con las dinámicas y complejidades inherentes a las costumbres y la realidad de los territorios locales^4^.

En este sentido, la ejecución del proyecto “Nós da Linha de Frente II” se llevó a cabo en tres fases: 1) conocimiento del grupo voluntario y alineación del servicio ofrecido con las necesidades identificadas en el escenario local; 2) apoyo para la ejecución del trabajo, que incluyó la divulgación del servicio, la creación de formularios de inscripción para la recepción y el manejo de la demanda, la articulación de espacios de supervisión técnica gratuita, la mediación entre la coordinación de grupos voluntarios y los órganos ejecutivos estatales, entre otros; y 3) construcción de un servicio en línea de formación multiprofesional para profesionales locales, con el fin de mantener la continuidad de la atención después de que concluya el servicio de voluntariado.

Este último, en particular, reflejó la evidente necesidad de cualificación de profesionales para actuar en situaciones de respuesta a períodos de crisis sanitaria en Brasil, reforzando la importancia de programas de formación que, en formato en línea, brindan un amplio acceso a la formación multiprofesional.

La propuesta del curso estuvo en consonancia con el estudio de Kabad *et al*.^5^, al concluir que, además de las acciones rápidas de asistencia, se necesitan estrategias de traducción del conocimiento capaces de producir síntesis y formación en configuraciones amigables, con un lenguaje simple y mensajes directos, para aquellos que forman parte de los equipos de gestión y trabajo.

La iniciativa de evaluar la ejecución de un *Curso básico de intervención terapéutica en situaciones de pérdida traumática y duelo*, desde un enfoque multiprofesional, surgió a partir de la necesidad de fundamentar científicamente las estrategias de respuestas rápidas en el marco de una crisis sanitaria. Si bien, por un lado, la modalidad en línea posibilitó el distanciamiento físico sin perder el contacto social, también profundizó las desigualdades preexistentes en el acceso a la educación^6^, especialmente aquellas mediadas por la tecnología.

Por lo tanto, este estudio presenta los resultados de la evaluación del curso de formación desde un enfoque multiprofesional, en el contexto de crisis sanitaria. Para ello, se recurrió al modelo de evaluación de cuatro niveles de Donald Kirkpatrick que, desde 1959, ha sido ampliamente aceptado en diversos tipos de organizaciones. La evaluación de los niveles de reacción, aprendizaje, comportamiento y resultados proporciona una perspectiva ampliada del proceso de formación y los resultados alcanzados^7^.

A su vez, dado el carácter teórico-práctico del curso, el modelo Kirkpatrick se presenta como una herramienta de evaluación viable para dimensionar el puente necesario entre formación y práctica.

La investigación se justifica por la necesidad de comprender los impactos de este tipo de iniciativas en la salud mental y la formación profesional de aquellos que ayudaron voluntariamente en la primera línea, así como para ampliar el conocimiento sobre las ramificaciones de la pandemia como fenómeno de alcance biopsicosocial, con demandas de atención a múltiples sectores y profesiones.

Kabad *et al*.^5^ mencionan la combinación del trabajo voluntario y colaborativo como una estrategia fundamental para formular algunos temas basados en experiencias de emergencias en salud pública y pandemias, con el propósito de reflexionar y contribuir a situaciones similares en el futuro. También se parte de la perspectiva de que el papel de la universidad pública, a través de la extensión universitaria, es la construcción de articulaciones entre instituciones y personas que, colectivamente, se han constituido como redes vivas en salud^3^ en el momento de respuesta a la crisis sanitaria en Amazonas. Frente a estas premisas, este estudio tuvo como objetivo evaluar el *Curso básico de intervención terapéutica en situaciones de pérdida traumática y duelo*, ofrecido en modalidad en línea por la Universidade do Estado do Amazonas, centrándose en aspectos de formación, aprendizaje, práctica y producción de resultados.

## MÉTODO

Se realizó un estudio transversal con un enfoque cuantitativo y cualitativo, de carácter descriptivo y exploratorio. En el enfoque cuantitativo, a través de estadísticas descriptivas, se describió el perfil de los participantes del curso, se calculó el índice de satisfacción y el impacto percibido por las personas que fueron atendidos por las y los profesionales voluntarios en formación. El enfoque cualitativo permitió el análisis de contenido relacionado con los conocimientos adquiridos y los cambios observados por las y los profesionales, así como el impacto del trabajo desde la perspectiva de las personas atendidas.

El *Curso básico de intervención terapéutica en situaciones de pérdida traumática y duelo* se llevó a cabo desde el 13 de abril al 1 de agosto de 2021, a través de la plataforma Zoom. Las clases se realizaron en modalidad en línea, con una propuesta expositiva-dialogada, y una duración de 3 horas cada una. Las referencias bibliográficas utilizadas fueron principalmente materiales científicos y recursos de intervención de autoría de las y los mediadores invitados (dada su experiencia en el tema), y también hubo material auxiliar de elección libre de cada docente. En la Tabla 1 se presenta el contenido del curso, la persona a cargo y su filiación institucional, el período y la bibliografía básica utilizada durante las clases del curso.

**Tabla 1:** Características del Curso básico de intervención terapéutica en situaciones de pérdida traumática y duelo. Manaus, Amazonas, Brasil, 2022.

Contenido del curso	Mediador	Institución	Período	Referencias bibliográficas*
Muerte y pandemia: repercusiones clínicas para el proceso de duelo	Profesora Doctora Maria Júlia Kóvacs (psicóloga)	Instituto de Psicología, Universidade de São Paulo	Abril, 2021	Kovacs MJ. Educação para a morte: Desafios na formação de profissionais de saúde e educação. São Paulo: Casa do Psicólogo; 2003.
Pandemia y condiciones psiquiátricas resultantes	Profesor Doctor Marcus Zanetti (médico psiquiatra)	Hospital Sírio-Libanês	Abril, 2021	Silva TF, Lopes JAS, Zanetti MV. Transtorno do estresse pós-traumático e COVID-19. In: Kovacs MJ, Pallotino ER, Aceti D, Ribeiro HG, (org). Luto e saúde mental na pandemia de COVID-19: cuidados e reflexões. Novo Hamburgo: Sinopsys; 2022.
Pérdida traumática y duelo: conceptos básicos y tipologías	Profesora Doctora Maria Helena Pereira Franco	Instituto Maria Helena Pereira Franco	Mayo, 2021	Franco MHP, Cogo AS, Andery MCR, Rigonatti V, Cruz GCQTV, Peixoto TC, Donno GV, Hernandes LR, Ohara ASM, Prizantelli CC, Guedes IAA, Piva NA, Silva M, (org). A experiência no suporte emocional a enlutados na COVID-19: intervenção on-line por psicólogos especialistas em luto. Curitiba: CRV; 2023. Franco MHP, Andery MCR, Luna IJ, (org). Reflexões sobre o luto: práticas interventivas e especificidades do trabalho com pessoas enlutadas. 2a ed. Curitiba: Editora Appris; 2022.
Proceso de duelo en la infancia y adolescencia: manejo terapéutico	Magíster Ariane Fernandes (psicóloga)	Hospital Universitário Onofre Lopes, Universidade Federal do Rio Grande do Norte	Mayo, 2021	Therense M, Perdomo SB, Fernandes ACS. Nós da linha de frente: diálogos sobre o ser da saúde no contexto da pandemia. Cadernos de Psicologia Social do Trabalho. 2021;24(2):265-278.
Asesoramiento terapéutico en modalidad virtual para familiares en duelo: posibilidades y límites	Magíster Cecília Rezende (psicóloga)	Instituto de Psicologia Entrelaços	Junio, 2021	Kovacs MJ, Pallotino ER, Aceti D, Ribeiro HG, (org). Luto e saúde mental na pandemia de COVID-19: cuidados e reflexões. Novo Hamburgo: Sinopsys; 2022.
Intervenciones terapéuticas para situaciones de pérdidas traumáticas y duelo	Magíster Érika Pallottino (psicóloga)	Instituto de Psicologia Entrelaços	Junio, 2021	Instituto Entrelaços. Projeto Vamos falar sobre o luto? [vídeo]. 2015. Disponible en: https://youtu.be/9y14iGqbO80.
Espiritualidad y religiosidad en los procesos de duelo	Profesora Doctora Geórgia Sibele Nogueira da Silva (psicóloga)	Universidade Federal do Rio Grande do Norte	Junio, 2021	Vasconcelos MO, Nogueira da Silva GS. A espiritualidade no lidar com a morte e o morrer na atenção primária à saúde. In: Paulino M, (org). Flores de Mandacarus: produções cultivadas no Mestrado Profissional em Saúde da Família da RENASF/UFRN. Natal: EDUFRN; 2021.

Fuente: Elaboración propia. *Durante el período de clases, algunas referencias no habían sido publicadas aún, dado que eran documentos creados específicamente para impartir las clases y otras se encontraban en proceso de publicación.

Se ofrecieron 70 plazas a profesionales voluntarios que habían participado en el proyecto “Nós da Linha de Frente” desde el inicio de la segunda edición y 30 plazas gratuitas para aquellos que quiseran sumarse al trabajo voluntario y dar atención gratuito a profesionales que actuaban en la línea del frente de la pandemia. Debido a la alta demanda del curso, se abrieron 20 plazas adicionales, sin especificidad de público objetivo.

### Fuentes de información y estrategia de recopilación de datos

La muestra se conformó con los datos secundarios de los formularios de evaluación del proyecto de extensión “Nós da Linha de Frente”, que fueron respondidos de manera voluntaria por los profesionales y pacientes atendidos al finalizar el proyecto. Los formularios de evaluación fueron elaborados por la coordinadora general del proyecto y la asistente becaria, siguiendo los elementos indicados en la bibliografía del área^7^.

Como criterios de inclusión, se tuvieron en cuenta las respuestas contenidas en la base de datos del proyecto “Nós da Linha de Frente”, en referencia al Formulario de Evaluación de la Experiencia del Voluntario y al Formulario de Evaluación de la Experiencia del Paciente. Como criterio de exclusión, se excluyeron las respuestas de personas que participaron y son investigadoras/es del proyecto de extensión.

Con un formato semiestructurado que incluía preguntas abiertas y cerradas, ambos formularios -“Formulario de Evaluación de la Experiencia del Voluntario” (para profesionales voluntarios) y “Formulario de Evaluación de la Experiencia del Paciente” (para personas atendidas) se enviaron por correo electrónico después de completar el curso. Se utilizó la plataforma Google Forms porque ofrece una hoja de cálculo con posibilidad de descarga y organización de la información recopilada.

El número de respuestas al “Formulario de Evaluación de la Experiencia del Voluntario” fue de 55 respuestas, equivalente al 50% del total de estudiantes participantes, y el número de respuestas al “Formulario de Evaluación de la Experiencia del Paciente” fue de 14 respuestas, equivalente al 50% del número de pacientes atendidos por las y los profesionales participantes.

La investigación fue sometida al Comité de Ética en Investigación de la Universidad del Estado de Amazonas y fue aprobada bajo el dictamen nº 5.287.816.

### Análisis de los datos

En cuanto al enfoque cualitativo de la investigación, que incluyó el contenido del formulario relativo a las preguntas abiertas y la revisión documental, se utilizó la técnica de análisis de contenido, adaptada para cumplir con la propuesta del presente estudio. Se realizó una exploración previa de las respuestas del formulario relacionadas con el curso, una lectura flotante de estas y la construcción de temas que indicaran posibles categorías^8^.

Luego, se seleccionaron las unidades de análisis, las cuales fueron categorizadas basándose en el marco teórico de Kirkpatrick^7^, cuyo modelo de evaluación del proceso formativo se realiza a partir de la medición de cuatro niveles: *reacción*, *conocimiento*, *cambio* e *impacto*.

El nivel 1 de evaluación fue la *reacción* del profesional voluntario y del paciente; el nivel 2, la evaluación del *aprendizaje* demostrado durante el proceso de formación; el nivel 3, los *cambios de comportamiento* generados por la participación en el curso y la asistencia a un proceso psicoterapéutico breve; y el nivel 4, los *resultados* en las acciones realizadas por las y los profesionales voluntarios en las personas atendidas.

En todas las etapas, las respuestas de ambos formularios se relacionaron y compararon mediante análisis cualitativo y cuantitativo. La Tabla 2 presenta los elementos que se evaluaron en cada nivel, a partir de la reproducción parcial del modelo elaborado por Santos *et al*.^9^.

Desde el enfoque cuantitativo, los datos de los formularios se analizaron utilizando estadísticas descriptivas. Los datos se estratificaron según la escala Likert, que midió el nivel de satisfacción de 0 a 10.

## RESULTADOS Y DISCUSIÓN

Para facilitar la comprensión de los datos, comenzamos con la presentación de un breve mapeo del perfil de las personas participantes del curso, recopilado en la primera sección del cuestionario. 

La participación presentaba particularidades en relación con las motivaciones y el contexto en el que se desarrolló, su estructuración, sus supuestos y los instrumentos pedagógicos. En línea con la iniciativa de respuesta a la crisis sanitaria, la acción surgió con el propósito de formar profesionales para la intervención terapéutica en situaciones de pérdidas traumáticas y duelo en el ámbito de la pandemia, de manera interprofesional. 

De las 55 personas que respondieron el instrumento de evaluación, el 47,28% participaron solo en la fase tres del proyecto “Nós da Linha de Frente II” y el 52,72% estuvieron presentes desde el principio, en la fase de formación de los grupos voluntarios.

En cuanto a la región de origen en Brasil, el 62,4% eran del sudeste, el 22,8% del norte, el 7,6% del nordeste y el 7,2% del sur. En un estudio que evaluó una formación en línea y abierta sobre la difusión científica en la pandemia de covid-19, la región sudeste también presentó una mayor concentración en la participación de la formación^10^.

Es importante destacar que en la región norte, el escenario de baja participación podría explicarse a partir de los resultados de una investigación que analizó las narrativas de las y los profesionales de la salud en Amazonas, quienes señalaron un agotamiento diario con la muerte, así como estrategias funcionales y disfuncionales adoptadas para enfrentar los problemas y mantener la salud mental^2^.

**Tabla 2:** Modelo Kirkpatrick de cuatro niveles: aspectos evaluados, categorías evaluativas y fuente de información y datos. Manaus, Amazonas, 2022.

Nivel de evaluación	Aspecto evaluado	Categorías
Reacción	Satisfacción con la formación del Curso básico de intervención terapéutica en situaciones de pérdida traumática y duelo, utilizando una escala tipo Likert de diez puntos (0 a 10) y una pregunta abierta sobre recuerdos significativos del proceso formativo.	"¿Cuál es el grado de satisfacción con el curso?" "¿Hay algún recuerdo significativo relacionado con el trabajo desarrollado por el proyecto que te gustaría compartir con nosotros?"
Conocimientos	Adquisición de conocimientos, desarrollo de habilidades relacionadas con el tema y cambios de actitud. Se analizaron los contenidos de las narrativas textuales de las preguntas abiertas.	¿Por qué decidiste ser voluntario en el proyecto “Nós da Linha de Frente”? ¿Qué beneficios encontraste al realizar el voluntariado en “Nós da Linha de Frente”? ¿Cuáles fueron los desafíos que enfrentaste al realizar el voluntariado en el proyecto “Nós da Linha de Frente”?
Comportamiento	Aplicación de los elementos destacados en el Nivel 2 en la ejecución del trabajo práctico. Se analizaron los contenidos de las narrativas textuales de las preguntas abiertas, relacionadas con la autopercepción del impacto del curso.	"Para ti, ¿cuál es el principal impacto causado por tu trabajo en el proyecto?"
Resultados	Acciones presentes en la atención que pueden estar relacionadas con los contenidos trabajados en el curso de formación. Se creó un cuestionario específico para las personas atendidas, compuesto por preguntas tipo Likert de cinco puntos y una pregunta abierta.	“En una escala del 0 al 10, ¿cuál es su nivel de satisfacción con el profesional que lo atendió?” “En una escala del 0 al 10, ¿cuál es el grado de conocimiento que el profesional que lo atendió tenía sobre el sufrimiento generado por el duelo?” “En una escala del 0 al 10, ¿cuál es el impacto que la atención psicológica generó en su proceso de duelo?” “En una escala del 0 al 10, ¿cuánto la atención psicológica promovió la salud mental para usted?”

Fuente: Elaboración propia.

Sin embargo, en un estudio sobre la experiencia de un curso de salud mental en el contexto del covid-19, con pueblos indígenas y a través de la enseñanza remota, mostró una mayor concentración de participación (41%) en la región norte de Brasil^11^.

En cuanto a la edad, el 20% tenía entre 18 y 30 años, el 44% entre 31 y 45 años, el 24% entre 46 y 60 años y el 12% tenía más de 60 años. Un estudio señaló datos similares, con relación al mismo rango de edad, en la participación en formación en línea sobre conocimientos de la pandemia de la covid-19^10^.

En cuanto al perfil profesional, el 22% tenía hasta un año de haberse graduado en sus áreas de actuación, el 27% entre uno y cinco años, el 16% entre seis y quince, el 31% entre dieciséis y treinta años, y el 4% estaba a menos de un año de la jubilación o ya la estaba disfrutando.

A través de un análisis cualitativo, se observó que la mayoría de los participantes se interesaron en el proyecto por la importancia de continuar su formación intelectual, así como por la prestación de servicios humanitarios frente a las noticias difundidas por los medios de comunicación. Del total de participantes, el 52,72% ya estaba trabajando en alguno de frente del proyecto “Nós da Linha de Frente”. Los demás (47,28%) se comprometieron a realizar el seguimiento de un paciente durante un período de tres meses. Después de eso, los voluntarios recibirían la certificación del trabajo voluntario de extensión, emitida por la Pro-Rectoría de Extensión de la Universidade do Estado do Amazonas.

### Evaluación de los cuatro niveles, a partir del modelo Kirkpatrick

#### Nivel 1: Reacción

En este nivel de análisis, se midió la satisfacción de las y los profesionales con relación al curso de formación ofrecido, utilizando para ello el cálculo del índice de satisfacción recopilado en una pregunta tipo Likert específica sobre este tema y una pregunta abierta sobre recuerdos significativos del proceso formativo.

De los 55 encuestados en la pregunta tipo Likert, el 7,3% evaluó con un grado de satisfacción de 8; el 14,5% evaluó con un grado de 9; y el 78,2% evaluó con un grado de 10. Estos resultados, de acuerdo con el nivel de reacción del modelo de los cuatro niveles^7^, revelan que el entrenamiento fue muy bien recibido por el grupo. Estos resultados indican convergencia entre este estudio y la bibliografía. Según Amorim *et al*.^12^ y Pereira^13^, el trabajo voluntario fomenta el bienestar social, emocional y psicológico, generando impactos positivos para la salud de los individuos; destaca valores relacionados con las preocupaciones humanísticas; promueve el desarrollo de habilidades, aprendizajes, conocimientos y preparación para una futura carrera profesional y la mejora de la satisfacción personal, la autoestima, el autoconocimiento y la formación de vínculos. Por lo tanto, se entiende que la reacción de satisfacción está asociada a estos componentes.

Las especificidades del curso que contribuyeron a este cuadro de reacciones fueron identificadas en las respuestas a la pregunta “¿Hay algún recuerdo significativo relacionado con el trabajo desarrollado en el proyecto que te gustaría compartir con nosotros?”. De estas respuestas, se desprende que el 32,72% indicó satisfacción por la posibilidad de construir colectivamente un trabajo con otros profesionales y recibir capacitación con disertantes motivados; el 25,45% también lo señaló a partir del feedback positivo que recibieron de las personas atendidas después de finalizados los tratamientos; el 20% consideró significativo el contacto con una experiencia de vida inédita proporcionada por la persona atendida; el 7,29% indicó una reacción positiva relacionada con la posibilidad de contribuir a la universidad pública y responder a las iniciativas insuficientes del Ejecutivo Federal; y el 14,54% no respondió.

La mayor incidencia de satisfacción relacionada con la experiencia de trabajo colectivo y con la participación de disertantes motivados se puede ejemplificar con los siguientes extractos de respuestas, identificados con la letra “P”, seguida del número correspondiente al orden de completado del formulario:

“*Me emocionó ver a otras profesionales listas para brindar atención, preocupadas por los demás y dando todo lo que pueden para realizar su trabajo. En este momento tan complicado, con demostraciones constantes de negacionismo, insensibilidad y maldad provenientes del presidente y de la población, es muy gratificante y acogedor ver a otras personas listas para ayudar.*” (P28)

“*En general, poder observar y percibir la entrega que numerosos profesionales hicieron de sí mismos y de su aparato técnico y humano en beneficio de las personas que son atendidas o capacitadas para la atención, a través de este proyecto.*” (P36)

“*La riqueza y diversidad de los formadores y de los profesionales que actúan como voluntarios. Ser un equipo de trabajo que abarca desde el norte hasta el sur, desde el este hasta el oeste. La motivación de quienes exponían también era algo emocionante.*” (P17)

“*Creo que los profesionales del curso tuvieron discursos sabios y de gran humildad. Realmente deseo que esto sea constante en el campo de la psicología*.” (P39)

Los relatos que surgieron en el nivel 1 de *reacción* indican que la experiencia parece haber sido un aporte para los profesionales voluntarios, especialmente a través de la organización del trabajo colectivo. En los fragmentos expuestos, expresiones como “otros”, “innumerables” y “fuerza de tarea” trazan esta noción de experiencia compartida.

Con el auge del tercer sector, se desarrolló una legislación específica, la Ley 9068/1998^14^, que organiza el servicio voluntario como una actividad sin remuneración financiera, con un amplio espectro de objetivos, tales como científicos, educativos, cívicos y/o de asistencia a la persona. Como polo activo, el proyecto de extensión reunió una gama de iniciativas, motivaciones y ofertas individuales, organizándolas. Al hacerlo, permitió el encuentro de colegas de profesiones, actuando como agente potenciador del trabajo.

Así, a pesar de haber sido un período de crisis aguda en el Amazonas, se observa la participación de la región y de la universidad como participantes activos en la red viva de cuidados de la salud, no como polo exclusivamente beneficiado o asistido, rompiendo con el patrón colonizador existente en los voluntariados entre regiones en Brasil.

Es importante destacar esta perspectiva porque, en general, la doble naturaleza del trabajo voluntario, al satisfacer las necesidades existenciales de ambas partes involucradas en la actividad realizada, suscita cuestionamientos críticos sobre la acción desarrollada. Una de las principales críticas es que, al satisfacer al voluntario en sus necesidades de respuestas existenciales, el voluntariado puede quitar la complejidad del fenómeno asistido, dándole una apariencia que enmascara las estructuras que permiten la perpetuación de los problemas sociales observados.

Para Bucci^15^, una de sus principales fuerzas del voluntariado sería la satisfacción de un amplio sector de la población por cubrir las necesidades básicas de un sector empobrecido, como un camino de autoayuda. Sin embargo, para Melo^16^, el mercado ha capturado el trabajo voluntario y, a partir de él, ha producido relaciones humanas que pueden estar distantes del debate de la jerarquía y opresión de clases. Así, autor y autora, respectivamente, exhiben contradicciones y resonancias con respecto al trabajo voluntario, que deben ser debatidas.

A partir de tales consideraciones, podemos acercarnos a la provocación de Bucci^15^, que señala la necesidad de construir una actuación solidaria que manifieste un compromiso más radical con las cuestiones observadas. La provocación del autor nos lleva a la discusión de la asistencia sanitaria desde el enfoque de los derechos humanos, que alerta sobre la necesidad de establecer movimientos consistentes y emancipadores que reafirmen y protejan el valor de la dignidad humana.

En términos de asistencia sanitaria, esto implica, según Oliveira *et al*.^17^, fomentar el acceso a servicios de salud e información de calidad, la no discriminación y la participación en las decisiones. Al parecer, la acción colectiva para ofrecer los servicios asistenciales voluntarios también involucró, según las declaraciones destacadas, la lectura del escenario político nacional, reflexiones sobre el compromiso ético-profesional y nociones sobre el ejercicio de la ciudadanía brasileña. Esto puede acercar la discusión del presente estudio a la provocación hecha por Bucci^15^, en relación con la solidaridad comprometida radicalmente con la transformación de estructuras que cristalizan fracturas ético-políticas en nuestro país.

#### Nível 2: Conocimiento

En este nivel de análisis, se evaluó el aprendizaje proporcionado por el curso de formación. Para ello, se analizaron las respuestas dadas a las preguntas sobre las motivaciones. El 40% de las respuestas mencionaron palabras relacionadas con la ayuda o contribución ante la situación de emergencia de otros; el 38,19% se refirieron a la iniciativa híbrida compuesta por ser útil y aprender; el 16,36% hicieron referencia a los aprendizajes que podrían aportar a la carrera profesional; y el 5,45% dio respuestas generalistas que dificultaron el análisis. De esta manera, el 54,57% de las y los participantes que estuvieron en el proyecto mencionaron como objetivo adquirir conocimientos. Haciendo una relación con la categoría anterior, se puede sugerir que la reacción positiva de satisfacción presentada por quienes asistieron al curso se debió, entre otras razones, a la adquisición de habilidades en respuesta a la crisis sanitaria, contribuyendo de esta manera al perfeccionamiento profesional.

Cuando se les preguntó sobre los beneficios recibidos al realizar el voluntariado, las respuestas que abordaron este aspecto mostraron que el 56,36% de quienes participaron fueron generalistas al referirse a los conocimientos adquiridos, enmarcando los contenidos de una manera global; el 27,28% indicaron las temáticas de duelo, atención en línea focalizada y características de la cultura amazónica; y el 16,36% enfatizaron beneficios no relacionados con la adquisición de conocimientos. Los siguientes relatos ejemplifican estos contenidos narrados:

“*La mayoría de las clases fueron buenas, y la supervisión también fue muy buena. Dejo mi reconocimiento y mis felicitaciones por la ejecución de un proyecto bien organizado, que no fue simplemente un voluntariado, realmente fue una formación/capacitación de calidad para aquellos que tenían dificultades o poca experiencia, según pude observar de algunos participantes. Algo que me llamó la atención durante esta pandemia fue el exceso de profesionales ofreciéndose como voluntarios (críticas con relación a esto debido a la desvalorización de nuestra categoría. No vi a otras categorías con tantos profesionales trabajando de forma gratuita como lo hizo la nuestra), sin siquiera tener control o supervisión de lo que estaba sucediendo. El proyecto hizo la diferencia. Realmente marcó la diferencia*.” (P4)

“*Oportunidad de acceder a personas que necesitaban ayuda y de hacer algo por el colectivo; ambiente de respeto, consideración, cuidado, solidaridad y cooperación mutua; grupo de apoyo emocional; grupo de supervisión; iniciativas para mejorar las técnicas de autocuidado y una mejor atención psicológica frente a las emergencias; certificado de participación en el proyecto*.” (P14)

“...*en mi caso, fue el intercambio regional, las diferencias de estas experiencias fueron de extrema importancia para mí*.” (P25)

Finalmente, en lo que respecta a la evaluación de los conocimientos adquiridos, también se analizaron los contenidos de la pregunta sobre los desafíos enfrentados en la ejecución del voluntariado. En este sentido, el 9,09% destacó conocimientos generalistas, sin especificar contenidos; el 25,46% afirmó haber enfrentado desafíos en la atención de pérdidas múltiples y duelo; el 38,18% se refirieron a las dificultades relacionadas con el uso de tecnologías y las regionalidades, como lo señalan los siguientes fragmentos:

“*Realizar atención en línea para personas de otra región*.” (P30)

“*Impacto emocional debido al gran número de muertes; gran demanda de atenciones; novedad en la dinámica de actuación debido a la pandemia y a la extensión y diversidad del territorio nacional*.” (P23)

En los porcentajes y en las declaraciones destacadas, se observa algo diferente en la relación caritativa-asistencialista que históricamente ha marcado las relaciones entre el norte, el nordeste y las demás regiones de Brasil. Según Souza y Ayres Pinto^18^, las representaciones de un pueblo miserable, hambriento, desinformado y sin capacidad para el trabajo se difundieron por todo el país, generando justificaciones para tolerar la exclusión de las regiones y eximiendo a la agencia del Estado en el proceso de opresión de los pueblos del norte y nordeste. Así, cuando las participantes resaltan la adquisición de conocimientos provenientes de un proyecto de extensión de una universidad pública de Amazonas, están presentando un eje de articulación interregional que hace justicia a las potencias y diversidades del norte de Brasil.

Otro dato interesante que muestran los resultados es que los contenidos más destacados en el proceso de adquisición de conocimientos fueron la asistencia a la muerte y el duelo. Según la bibliografía^19^, la formación en salud se basa en el modelo biomédico, cuyo guion enfatiza la cura como éxito técnico y la muerte como fracaso. Este escenario impermeabiliza las matrices curriculares para contenidos relacionados con la asistencia al proceso de muerte y duelo, produciendo, según señala Franco^20^, profesionales que se gradúan y experimentan duelos no reconocidos y no vividos, desarrollando cuadros agudos de sufrimiento mental, desinversión en el autocuidado y debilitamiento de los vínculos entre el profesional y el usuario. Por ende, desde su nomenclatura, el *Curso básico de intervención terapéutica en situaciones de pérdida traumática y duelo* se propuso ofrecer conocimientos que acerquen a los profesionales de la salud a la discusión sobre la asistencia a la muerte y el duelo.

Finalmente, el contacto con realidades regionales diferentes, que visibilizó la diversidad del territorio nacional, se constituyó como un espacio de adquisición de conocimiento. Expresiones como novedades e intercambios regionales convergen con la reflexión de Alcaraz^21^ sobre cómo las diferencias median las pluralidades.

En este sentido, la viabilización de encuentros entre personas de regiones diferentes parece haber permitido reflexiones sobre la heterogeneización de vivencias y la importancia de experiencias que se interpongan a la dominación cultural.

#### Nivel 3: Comportamiento

En esta sección, se buscó evaluar lo que hay de diferente en el comportamiento de los participantes después del curso. En este aspecto, se revela que, entre ellos, la participación suscitó transformaciones en la forma de analizar sus prácticas profesionales y personales, así como en el modo de actuación social, en pro de la promoción de la salud mental:

“*Búsqueda de nuevos sentidos de vida.*” (P16)

“*Proporcionar apoyo en el momento de necesidad, saber escuchar.*” (P21)

“*Volverse más humano y democratizar la psicología.*” (P43)

“*Aprendizaje, crecimiento personal y profesional.*” (P47)

Estos resultados están en sintonía con Scorsolini-Comin *et al*.^22^, quienes reflexionan sobre el uso del modelo de Kirkpatrick^7^ en la evaluación de la educación a distancia. Su estudio revela registros de que el proceso de toma de conciencia por parte de quienes participan en la formación dependerá, incluso, del modo en que se construyan las herramientas de aprendizaje, facilitando o dificultando el proceso de asumir la autoría con relación a su propio conocimiento. En este sentido, la principal herramienta de aprendizaje que medió la formación fue el protagonismo en la atención clínica y la articulación con la teoría durante las clases.

Otro aspecto importante a destacar es la noción de concientización de Paulo Freire^23^ que aportó el curso, así como la conciencia expresada a través del compromiso de transformación^23^ que asumieron las y los participantes.

Entre estas transformaciones de comportamiento expresadas en los relatos, es importante destacar la alteridad como un elemento concreto en el cambio de comportamiento, a modo de ejemplo, se describe otro testimonio:

“*Fue un intercambio que me permitió entender al otro desde una perspectiva diferente, antes no trabajaba el duelo y era un tema distante para mí, hoy estoy muy comprometida con este asunto y esto se convirtió en algo que se incorporó a mi vida y estudios*.” (P25)

En su testimonio se observa que se activa la noción de “intercambio” como facilitador del ejercicio de la alteridad e impulso a la interacción. Según su percepción, la formación se basó en aspectos de la *educación dialógica*, que corresponde a un encuentro de personas, a la mutualidad inseparable entre el educador y el aprendiz en el proceso de enseñar-aprender^24^. Además, la formación le proporcionó cambios no solo en comprender y dialogar con las diferencias, sino que el curso también pasó a formar parte del área de interés y actuación de la persona participante. Por lo tanto, se evidencia la incorporación del conocimiento a la práctica profesional y de estudios.

El trabajo de Scorsolini-Comin *et al*.^22^ señala que cuanto más el estudiante puede desempeñar un papel protagónico en una formación, tiende más a desarrollar su sentido crítico, alimentando la adopción de mejores estrategias didácticas y mejores evaluaciones del entrenamiento.

En este sentido, el curso, según las declaraciones analizadas, fue un momento que posibilitó procesos de transformación de vidas, ya sea a nivel personal o técnico-profesional, como se desprende de este otro fragmento:

“*Formación/preparación de estudiantes y profesionales para actuar con eficacia en el momento actual y en los que están por venir*.” (P55)

Las narrativas, a su vez, evidencian dos dimensiones existentes de cambio de comportamiento, desplegadas después del curso. La primera, que se analizó como afectivo-social, es la producción de cambios en los sentidos de la vida, el saber acoger en un momento de necesidad, escuchar, volverse sensible a las diferencias del otro; y la segunda, denominada técnico-profesional, definida mediante la producción de comportamientos de compromiso con el tema estudiado, la identificación como área de especialización y la preparación para la actuación concreta, teniendo en cuenta la carga horaria práctica del curso. Estas dos dimensiones parecen ser elementos potentes al estar presentes en formaciones durante períodos de crisis sanitarias.

#### Nivel 4: Resultados

Al evaluar el nivel de *resultados*, derivados de los cambios de comportamiento ocurridos después del entrenamiento, se observó que las narrativas se centraron en la idea de saber 

ejecutar una respuesta en situaciones de crisis sanitaria, como se puede ver en la Tabla 3.

**Tabla 3:** Resultados producidos por los cambios de comportamiento después del entrenamiento. Manaus, Amazonas, 2022.

Impactos	Narrativas de los participantes
Manejo clínico individual	"Actuación profesional y capacidad para aplicar los conocimientos adquiridos sobre duelo y ayudar en el dolor de las personas que están de luto. Posibilidad de atención en línea." (P2)
	"Haber ayudado a algunas personas a reorganizarse psíquicamente y seguir con sus vidas. En mí, haber escuchado tantos dolores similares de historias de vida tan diferentes." (P3)
	"La acogida que podemos brindar evita mayores traumas de soledad en este momento de Covid y duelo" (P6).
Manejo clínico ampliado	"Ayudar a una familia que está teniendo mucha dificultad para reconocer los sentimientos en este momento." (P7)
Instrumentalización	"He recibido muchas solicitudes de personas que enfrentaban crisis de ansiedad y síntomas depresivos. Fue importante brindar psicoeducación a estas personas y enseñarles herramientas para que pudieran estabilizarse emocionalmente durante una crisis." (P8)
Saber disminuir el sufrimiento	“Abrigar, ser abrigo.”(P11)
	"Rescate de la esperanza y la gratitud." (P12)
	"Aliviar, aunque sea un poco, los dolores que estamos experimentando en esta pandemia."(P15)
	"Receptividad ante el dolor en el momento crítico."(P23)
	"El acogimiento y la escucha a personas que estaban sufriendo. El dolor no cesa, pero es posible mirarlo." (P24)
	"Proporcionar un espacio acogedor para las personas en duelo y la posibilidad de elaborar las pérdidas para aquellas que no tienen condiciones financieras y acceso a los servicios de salud mental."(P30)
	"Estar presente, soportar el dolor con la persona." (P35)
Trabajar colectivamente	"Juntos somos más fuertes." (P56)
	"Matriciamiento[brindar apoyo matricial] de mis colegas de trabajo sobre la temática y potenciar los procesos de cuidado en salud de los pacientes y familiares." (P32)
	"Recibí mucho más de lo que esperaba poder contribuir. Me di cuenta de mi vocación para el voluntariado." (P14)

Fuente: Elaboración propia.

Es importante destacar que los participantes nunca habían enfrentado una situación de pandemia y, en su formación inicial o práctica, no tenían experiencia con muertes y personas en duelo en el contexto de una crisis sanitaria. Después del curso, pudieron hacer algo diferente o nuevo debido a la capacitación. Este hacer algo se caracterizó por el manejo clínico individual y ampliado, instrumentalizando el perfeccionamiento de prácticas comunes en la formación en psicología, como la psicoeducación, además de la conducción y el apoyo en situaciones de sufrimiento en catástrofes.

En este sentido, en un estudio sobre la experiencia de una capacitación nacional de emergencia en “Salud mental y atención psicosocial en covid-19”, se identificó que la formación académica de los profesionales que trabajan en situaciones de pandemia aún es un campo reciente de prácticas e investigación en Brasil^25^. En esa dirección, otra investigación^26^ reveló que, en los planes de estudio de la gran mayoría de los cursos universitarios así como en los programas de las asignaturas relacionadas con las políticas públicas de salud, hasta el momento, los temas de desastres y pandemias no se ofrecen en sus currículos.

La formación generó el sentimiento de unidad para enfrentar las consecuencias de la crisis sanitaria, entendida como un trabajo colectivo. El estudio de Kabad *et al.*^5^, sobre la experiencia del trabajo voluntario y colaborativo en salud mental y atención psicosocial durante la pandemia de covid-19, concluyó que, para las y los profesionales de la salud, reconocer la singularidad del escenario pandémico -la muerte, la enfermedad o enfermar, el aislamiento social, las pérdidas de ingresos y del empleo, las deudas y las incertidumbres sobre el futuro como factores de impacto en la salud mental- es crucial para determinar la búsqueda de conocimiento oportuno y en tiempo hábil. El mismo estudio señaló que combinar el trabajo voluntario y colaborativo con la experiencia, es decir, la posibilidad de reunir a un grupo de profesionales que nunca han trabajado juntos pero que se mostraron comprometidos en enfrentar la pandemia con sus conocimientos y experiencias académicas, puede ser la energía fundamental para constituir el trabajo colectivo y voluntario^5^. 

Entre las declaraciones sobre los resultados de la formación, P32 se destacó por utilizar un término común en el ámbito de la salud mental: el matriciamiento. Este término reitera los aspectos de lo colectivo como un impacto que produjo la formación. Por definición, es la comprensión de que la producción de salud se realiza a través de un proceso de construcción compartida entre equipos de profesionales, con el fin de tratar las dificultades de una persona mediante una propuesta de intervención pedagógica y terapéutica conjunta. También es destacable el sentimiento atribuido por P14 a la formación como un elemento necesario en la práctica del voluntariado. Es importante destacar que, mediante la formación, aunque no sea el enfoque principal, se ha vuelto posible comprender, según el estudio de Silva *et al*.^27^, la importancia del apoyo matricial como una herramienta de cogestión que potencia la participación activa de los trabajadores en los procesos de trabajo.

En cuanto a las catorce respuestas de los pacientes atendidos por el proyecto, en relación con el nivel de satisfacción con el profesional que llevó a cabo la atención, el 64,29% dio la calificación máxima; el 21,43% dio una calificación entre 7 y 9; y el 14,28% dio una calificación entre 0 y 1. En cuanto al grado de conocimiento que el profesional tenía sobre el sufrimiento causado por el duelo, el 64,29% dio la calificación máxima; el 14,28% dio una calificación entre 7 y 8; el 21,43% dio una calificación entre 0 y 5.

En relación con el impacto que el acompañamiento psicológico generó en el proceso de duelo, el 57,16% dio la calificación máxima; el 14,28% dio una calificación entre 8 y 9; y el 28,56% dio una calificación entre 0 y 5. Por último, con relación a cuánto el acompañamiento psicológico promovió la salud mental, el 50,02% dio la calificación máxima; el 21,42% dio una calificación de 9; y el 28,56% dio una calificación entre 0 y 5.

Según Espiridião^28^, cuando las y los profesionales de la salud prestan atención al proceso comunicacional no verbal, están disponibles para esperar el tiempo de expresión verbal e involucrados en comprender la perspectiva experiencial, las personas atendidas se sienten más cómodas y confiadas para hablar sobre sus problemas, y muchas veces perciben que esto facilita su resolución. En esta misma línea, Gomes *et al*.^29^ señalan que los pacientes tienden a sentirse más satisfechos. Por lo tanto, se entiende que la protección de la dignidad humana -denominada humanización de la asistencia- puede ser comprendida como una medida terapéutica para el manejo del sufrimiento.

## CONSIDERACIONES FINALES

Este estudio destaca la importancia y el potencial de los cursos de perfeccionamiento que entrelazan el eje académico con la perspectiva de transformación de las prácticas, ofrecidos en modalidad en línea. A través de la evaluación, se puede afirmar que el *Curso básico de intervención terapéutica en situaciones de pérdida traumática y luto* fue exitoso en sus objetivos.

Al analizarlo, desde su concepción hasta los resultados descritos, fue posible dimensionar el alcance de sus objetivos como proceso de formación y proyecto de extensión. Este curso buscó contribuir con una formación cualificada frente a la demanda aún no experimentada por los profesionales, que fue la pérdida y el duelo debido a la pandemia de covid-19.

Habiendo formado a 98 estudiantes, proporcionó a las personas egresadas los elementos que resultaron en acciones y reflexiones transformadoras de la práctica. Estos, a su vez, representan la base para la formación en emergencias en salud pública y pandemias, que exigen no solo estrategias de traducción del conocimiento, sino principalmente rapidez para proporcionar evidencias a los gestores y profesionales involucrados en la creación de respuestas a la sociedad.

Otro elemento importante es el trabajo voluntario realizado a través de las intervenciones psicológicas. En su momento, estuvo impregnado de críticas, tensiones y flexibilidades que no pueden resumirse en un solo estudio. Entre las reservas, estaba la idea de que estas intervenciones podrían suprimir la obligación del gobierno de contratar profesionales de salud mental para el cuidado de la población.

Sin embargo, la evaluación positiva del curso de formación multiprofesional del proyecto Nós da Linha de Frente sugiere que, a partir de él, se construyeron redes vivas en salud durante la pandemia de Covid-19, con la generación de indicadores que delinean la construcción de esta experiencia.

Además, a través del proyecto y del curso, se considera que los modos y caminos de cómo el Amazonas produjo formación y acceso a la salud no fueron fijos, previsiblemente estables, sino que se forjaron en una dinamicidad que, metafóricamente, remite al ciclo del agua, en medio de la mayor cuenca hidrográfica del mundo. Es decir, la dinámica de las articulaciones estuvo en constante movimiento y transformación, de acuerdo con las necesidades surgidas del propio contexto.

Por lo tanto, se señala que las urgencias y emergencias de la región amazónica incorporaron otros aspectos que van más allá del caso clínico o de lo instituido formalmente, dirigiendo los flujos en la construcción de formas de cuidado en cada situación. Así, fue atravesado y marcado por la producción del cuidado a partir de sus redes vivas, actuando fuera de los servicios formales, con múltiples puntos de conexión.

Siguiendo el modelo de los cuatro niveles, se puede entender que el trabajo colectivo y el desempeño de las y los docentes que legitimaba la importancia de la causa voluntaria, fueron elementos que contribuyeron a la reacción de satisfacción de los participantes.

En cuanto a los conocimientos adquiridos, los temas principales comprendieron aspectos relacionados con la asistencia en situaciones de muerte y duelo. Los comportamientos derivados del curso se clasifican en los campos considerados aquí como socioafectivos y técnico-profesionales.

En relación con el impacto, hubo una prevalencia de satisfacción entre los pacientes atendidos, así como la identificación de cambios causados por la capacitación, como el manejo clínico individual y ampliado, la instrumentación y mejora de prácticas, como la psicoeducación, además de la conducción y el apoyo al sufrimiento en situaciones de catástrofe.

De este modo, el estudio a la adaptación y aplicación del modelo de cuatro niveles, que puede ser útil para difundir un enfoque metodológico para la evaluación de resultados de cursos con el mismo contexto de emergencias y crisis.

La investigación presentó como limitación el hecho de que las preguntas de los formularios fueron elaboradas y proporcionadas antes de decidir utilizar el modelo de Kirkpatrick, lo que afectó tanto la orientación de la aproximación a los elementos de cada nivel como la aparición de respuestas más detalladas sobre las características, recursos e impactos del curso evaluado.

En cuanto a los resultados e impactos observados, se concluye que el curso puede ser simbolizado como un lugar de ruptura de la lógica colonizadora entre el eje sur-sudeste y el norte de Brasil, ya que la oferta de formación de calidad contribuyó activamente a la experiencia de las y los profesionales voluntarios. Este factor se debió a la inclusión de conocimientos poco explorados dentro de la formación general de los cursos de grado en salud y a la realización de intercambios de conocimientos y culturas entre las regiones.

En última instancia, este estudio sirvió como un registro del trabajo realizado por el proyecto “Nós da Linha de Frente”, edición 2021, que motivó el diálogo entre la bibliografía sobre universidad pública, voluntariado, salud colectiva y derechos humanos, en medio de una crisis de salud global.
